# Advancing sustainable practices with *Paenibacillus polymyxa*: From soil health to medical applications and molecular engineering

**DOI:** 10.3934/microbiol.2025016

**Published:** 2025-05-19

**Authors:** Imen Zalila-Kolsi, Ray Al-Barazie

**Affiliations:** Faculty of Medical and Health Sciences, Liwa College, Abu Dhabi P.O. Box 41009, United Arab Emirates

**Keywords:** *Paenibacillus polymyxa*, enzymes, biocontrol, genetic manipulation, protein expression, sustainable application

## Abstract

*Paenibacillus polymyxa* is a multifaceted bacterium with widespread applications in agriculture, environmental management, medicine, and industry. In agricultural settings, it plays a crucial role in soil enhancement, plant growth promotion, and natural pathogen control, reducing the need for chemical interventions. Additionally, *P. polymyxa* exhibits promising potential in medical applications by aiding in infection prevention and supporting gastrointestinal health. In the realm of environmental management, this bacterium contributes to pollution remediation through biodegradation processes. Industrially, *P. polymyxa* is involved in producing enzymes, biofertilizers, bioplastics, and platform chemicals, offering sustainable alternatives that underscore its importance in driving sustainability initiatives. Despite these valuable attributes, widespread utilization of bioresources derived from naturally occurring *P. polymyxa* has been hampered by limited genetic manipulation capabilities and tools. In this comprehensive analysis, we aimed to provide a thorough understanding of *P. polymyxa*'s characteristics, genetic resources, and metabolic capabilities, while highlighting its potential as a versatile platform for protein expression, metabolic engineering, and synthetic biology. We delved into the diverse sustainable applications of *P. polymyxa* in these domains, emphasizing its benefits, challenges, and future outlook in advancing sustainable practices. Furthermore, we underscore the critical need for continued research and development of advanced engineering techniques and genetic editing technologies tailored specifically for this bacterium.

## 1. Introduction

Since its discovery in 1880, *P. polymyxa* has undergone several name changes. Initially identified as *Clostridium polymyxa*, its classification was revised to *Bacillus polymyxa* in 1889 due to its rod-shaped cells [1]. In 1993, a new genus, *Paenibacillus*, was established through comparative 16S rRNA sequence analysis of the *Bacillus* genus, leading to the current designation of *P. polymyxa* [2]. *P. polymyxa* is commonly found in the rhizosphere and as an endophyte within plants. It is less frequently observed in marine sediments or fermented products. This bacterium is often harnessed for its ability to combat various pathogens that impact plants, humans, and animals. The endospores of *P. polymyxa* exhibit remarkable resilience, enabling them to withstand harsh environmental conditions such as high temperatures, biocides, pressure, and UV radiation. This durability enables the endospores to survive processes like pasteurization and persist in industrial equipment [3]–[5].

*P. polymyxa* synthesizes an array of compounds with biological activities, such as the lipopeptide polymyxin, fusaricidins, paenilipoheptin, paenilan, tridecaptin, 2,3-butanediol, and acetoin, which exhibit potential for combating multidrug-resistant infections and various microbial pathogens in plants and humans [6],[7]. These inherent capabilities position *P. polymyxa* as a model organism for sustainable health management in plants, humans, and animals under the One Health framework [8]. Despite its advantageous properties, the full utilization of bioresources derived from naturally occurring *P. polymyxa* has been hindered due to the lack of adequate tools and methods for genetic manipulation [9]. Advanced techniques and tools for genetic manipulation are needed to fully unlock the potential of this versatile bacterium in various applications.

Advancements in genetic manipulation techniques for *P. polymyxa* continue to progress, encompassing the utilization of shuttle plasmids, various promoters, and CRISPR genetic tools [10]. Metabolic engineering for the production of biocomposites has also been used. These techniques have been applied to enhance the production of biocomposites, such as 2,3-butanediol, from renewable resources [11]. Furthermore, the mechanisms for genetic transformation are evolving, with methods such as penicillin-assisted transformation, electroporation, and magnesium amino acid-assisted transformation being increasingly employed. The ongoing development of genetic manipulation tools, including technologies like homologous recombination and the CRISPR editing system, has positioned *P. polymyxa* as a promising microbial chassis for biomanufacturing. This expanded toolkit broadens the bacterium's applications, enabling its use in industrial enzyme production, bioremediation, bioadsorption, surfactant synthesis, and the production of antibacterial compounds [12].

Furthermore, *P. polymyxa* exhibits favorable characteristics for laboratory cultivation, as it can be easily cultured on agar media, is amenable to genetic modification, and has its entire genomic sequence readily accessible [13]. These combined attributes make *P. polymyxa* a versatile and promising organism for biotechnological applications. Numerous research studies have highlighted the potential of omics technologies in enhancing the applications of *P. polymyxa* in agriculture, medicine, and industry, particularly for mitigating interkingdom diseases. Pangenome examination of *P. polymyxa* strains offers an in-depth investigation of *P. polymyxa* strains, emphasizing their capability in generating antibiotics and secondary metabolites [14]. The isolation, identification, and characterization of *P. polymyxa* CR1 highlight the advantageous properties of *P. polymyxa* CR1, such as its uses in biopesticides, biofertilizers, and biofuel generation [15]. Genomic diversity in *P. polymyxa* investigates the genomic variation among *P. polymyxa* strains and their ecological effects [16]. These investigations have delved into the latest insights related to *P. polymyxa*, encompassing its role in biocontrol for disease management in plants, humans, and animals, genome analysis, antibiotic synthesis, and mechanisms that facilitate plant growth [17]. The findings from these studies underscore the diverse applications and promising potential of *P. polymyxa* across sectors, emphasizing its significant contribution to sustainable practices and disease management strategies.

*P. polymyxa* exhibits the capacity to produce a diverse array of physiologically active compounds, including phytohormones, antibiotics, enzymes, volatile organic compounds (VOCs), and exopolysaccharides (EPS) [18]–[22]. The wide range of bioactive compounds synthesized by *P. polymyxa* has led to the exploration of numerous applications in the agriculture and food sectors [23]–[25].

In agricultural contexts, the utilization of *P. polymyxa* revolves around two major aspects: Promoting plant growth and controlling plant diseases through biocontrol methods. The bacterium employs various mechanisms to support the growth and resilience of host plants, including nitrogen fixation, synthesis of phytohormones, solubilization of inorganic mineral phosphate, iron acquisition, and induction of systemic resistance [26]–[29]. These multifaceted activities underscore the potential of *P. polymyxa* as a valuable resource for sustainable agriculture practices and disease management strategies. *P. polymyxa* showcases promise as a sustainable agriculture option, serving as both a fertilizer and pesticide to enhance soil health and meet agricultural requirements [30]–[32]. The bacterium demonstrates antagonistic properties by producing a variety of compounds, including polypeptins, gatavalin, jolipeptin, fusaricidin, polymyxin, 2,3-butanediol, and acetoin, which exhibit efficacy against bacteria and fungi [33],[34]. Additionally, these antimicrobial compounds have the potential to provide protection against insect herbivores and plant pathogens by inducing a hypersensitive defense response in the host, a phenomenon known as induced systemic resistance [27]. This multifaceted functionality positions *P. polymyxa* as a versatile tool in sustainable agriculture practices, offering solutions for soil enrichment and pest management in an environmentally friendly manner.

In this paper, we provide a comprehensive review of the recent advancements in the applications of *P. polymyxa* across sectors, including industry, medicine, agriculture, and environmental management, as well as discussions on metabolic engineering and protein expression systems. Furthermore, we address the barriers that hinder the widespread utilization of this strain. Researchers seeking further insights into *P. polymyxa* and its diverse applications can refer to this review as a valuable resource.

## 2. The importance and sustainable applications of the *P. polymyxa*

**Figure 1.**
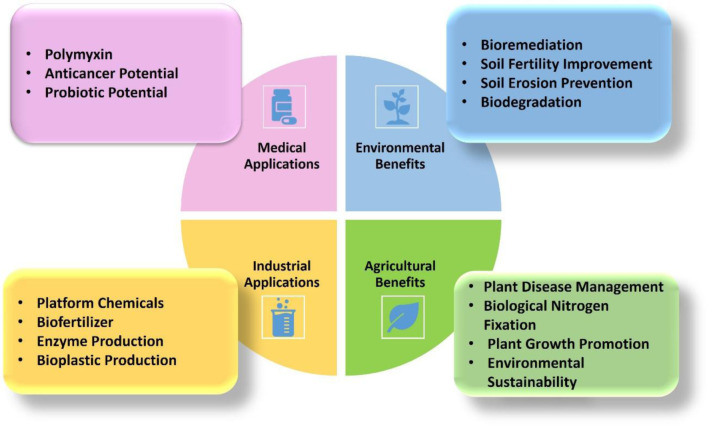
Sustainable application of *P. polymyxa* for the production of industrial chemicals or enzymes, agriculture, medicine, and the environment.

*P. polymyxa*, a rhizobacterium, has demonstrated significant utility across multiple sectors, including industry, agriculture, and medicine [16]. This bacterium is utilized for the production of valuable industrial compounds and antimicrobial agents, in addition to serving as a biofertilizer that enhances soil quality, boosts crop yields, and presents other advantageous applications [5] (Figure 1). In the subsequent sections, we delve into the diverse benefits and applications of this versatile bacterium.

### 2.1. Industrial application of *P. polymyxa*

*P. polymyxa* has demonstrated significant potential for the production of biofertilizers. Research has highlighted its capabilities, such as methane consumption and plant growth promotion, making it a promising candidate for this application [5]. Studies on *P. polymyxa* MaAl70 have shown its ability to consume methane and exhibit plant growth-promoting features, including the solubilization of essential nutrients like phosphorus, zinc, and potassium, atmospheric nitrogen fixation, and production of indole acetic acid, a key growth regulator in plants [35]. Similarly, research by Dal'Rio et al. (2024) on *P. polymyxa* CAPE238 revealed its positive impact on the germination speed and growth of *Tropaeolum majus* L. seeds [36]. These findings suggest that *P. polymyxa* has the potential to be harnessed for the development of biofertilizers that can effectively promote plant growth, serving as a sustainable alternative to conventional chemical fertilizers.

In the agricultural sector, the presence of plant fungal diseases and pests poses a significant challenge to crop cultivation, necessitating the use of antifungal agents. To mitigate the potential adverse effects of conventional chemical pesticides on human health and the environment, biocontrol agents have emerged as a more sustainable and environmentally friendly alternative. Strains of *P. polymyxa* have been successfully commercialized as biocontrol agents against fungal phytopathogens and pests [37].

One example is the strain *P. polymyxa* PKB1, which serves as an effective biocontrol agent by producing peptide antibiotics that combat a range of phytopathogens such as *Sclerotinia sclerotiorum*, *Rhizoctonia solani*, and *Fusarium avenaceum* [37]. By leveraging the biocontrol capabilities of *P. polymyxa*, farmers can effectively manage fungal diseases and pests in a sustainable manner while reducing reliance on chemical pesticides.

*P. polymyxa* is known to produce a diverse array of enzymes that hold significance in both the agricultural and industrial sectors. These enzymes include proteases, cellulases, xylanase, lipase, and pectinase [38]. Among these enzymes, glucomannanase plays a crucial role in the breakdown of konjac glucomannan (KGM), a polysaccharide with various applications in the pharmaceutical and food industries. Oligosaccharides derived from KGM have exhibited potent biological activities, including antitumor effects, in the field of life sciences [39]. Research by [39] identified the gene *ppglu*B responsible for encoding glucomannanase in *P. polymyxa* strain 3-3 and confirmed its enzymatic activity. This highlights the enzymatic potential of *P. polymyxa* and its ability to produce valuable enzymes with applications in various industries, underscoring the importance of exploring the enzymatic capabilities of this bacterium for future biotechnological advancements.

The experiments conducted have demonstrated that the identified gene can serve as a sustainable enzymatic hydrolysis technique for producing KGM oligosaccharides with diverse applications. On the other hand, lignin, an aromatic polymer crucial for plant structure, plays a vital role in protecting and supporting plant growth, and is widely utilized as a biofuel source [40]. However, accessing lignin from agricultural waste can be a challenging task. Degrading lignin is essential for enhancing its utility in various applications, such as enhancing the nutrient content of biological fertilizers and improving the flexibility of paper products [15]. One effective approach to decomposing agricultural waste, specifically lignin, is to leverage microorganisms capable of breaking down lignin through the production of specific enzymes [41]. This biological method offers a promising avenue for enhancing lignin degradation and utilizing this valuable compound effectively in various sectors. A study showcased that *P. polymyxa* strain 26 can produce ligninolytic enzymes, specifically laccase, lignin peroxidase, and manganese peroxidase. These enzymes play a significant role in enabling the bacterium to utilize lignin as its primary carbon source. This breakthrough highlights the potential for leveraging these ligninolytic enzymes in *P. polymyxa* for the degradation of agricultural waste, with promising implications for applications in the industrial sector [42].

Microbially induced corrosion has been recognized as a significant environmental factor influencing the cleaning and disposal of specific environmental waste. An intriguing study revealed that exposing glass to the *P. polymyxa* SCE2 biofilm for three months led to alterations in its chemical structure, indicative of microbially induced corrosion on the solid surface. This finding demonstrates the potential for utilizing microbial processes, such as those involving *P. polymyxa*, for environmental cleanup purposes [43].

In industrial biotechnology, microorganisms like bacteria play a crucial role in synthesizing valuable compounds. One noteworthy industrial compound produced through microbial processes is 2,3-Butanediol (2,3-BDL). This compound serves as a fuel additive with diverse industrial applications, including its use as an octane booster and in the production of solvents essential for synthesizing gums, resins, and paraffin wax [44]. Additionally, 2,3-BDL is involved in the manufacturing of rubber, plastics, natural flavorings, printing inks, perfumes, softening agents, and various other industrial products [45]. The synthesis of 2,3-BDL through bacterial fermentation has been established, with current producers being pathogenic bacteria such as *Klebsiella* and *Enterobacter*. However, the need for safer and more efficient alternatives led to the exploration of non-pathogenic bacteria like *P. polymyxa*, known for its ability to utilize a broad range of carbon sources, for the production of this valuable compound [46]. The efficiency and possible industrial uses of these processes have been highlighted by research that shows that *P. polymyxa* PM 3605 can produce (2R,3R)-butanediol from crude glycerol supplemented with sugarcane molasses [47],[48].

Despite the potential benefits, utilizing bacteria for the fermentation process presents challenges, particularly in achieving high production yields, a crucial industrial parameter. One strategy involves limiting oxygen in the fermentation process. Research shows that oxygen-limited circumstances can greatly increase yields and decrease the creation of byproducts, underscoring the significance of oxygen management in optimising the manufacture of 2,3-butanediol [49]. In a study involving anaerobic culture of *P. polymyxa* B-4317, the anaerobic fermentation of sugars was conducted, leading to improved production yields of 2,3-BDL. However, further evaluation of these methods is necessary to optimize the process effectively [50]. The study by Tinôco and Freire (2023), which examines the scaling-up of 2,3-butanediol production by *P. peoriae* NRRL BD-62 utilizing a constant oxygen transfer rate-based method, is another illustration of work in this area [51]. This research underscores the potential of utilizing non-pathogenic bacteria like *P. polymyxa* for industrial biotechnology applications such as 2,3-BDL production. Joshi et al. (2025) focused on optimizing the manufacturing process for *P. polymyxa* ATCC842 to produce 2,3-BDL. The study involved immobilizing the bacteria using chitosan-coated carrageenan beads. This novel approach has been shown to enhance 2,3-BDL production in a cost-effective manner, offering a sustainable alternative for industrial applications by harnessing the capabilities of *P. polymyxa* [46]. This innovative method has the potential to pave the way for more efficient and environmentally friendly production processes in the industrial sector.

Antimicrobial agents produced by rhizobacteria have been associated with promoting plant growth and offering a potential sustainable alternative for combating microbial infections, particularly considering the escalating concern surrounding antibiotic resistance [52]. In a study by Cho et al. (2020), the genes responsible for the plant growth-promoting (PGP) and antimicrobial activities of *P. polymyxa* strain TH2H2 were identified. The study revealed the presence of genes encoding for various antimicrobial agents, including fusaricidin (an antifungal agent) and antibacterial agents tridecaptin and polymyxin within this particular strain [53]. This research sheds light on the genetic basis underlying the antimicrobial and plant growth-promoting capabilities of *P. polymyxa*, emphasizing its potential utility in agriculture and as a sustainable approach for addressing antimicrobial resistance concerns in human health. In a study comparing two *P. polymyxa* strains, DSM32871 and M1, their production of antimicrobial agents was investigated. The research revealed variations in the types of antimicrobial agents produced by each strain. Strain DSM32871 produced polymyxin E and polymyxin P, whereas strain M1 only produced polymyxin P. Additionally, both strains exhibited the production of fusaricidins and tridecaptins, with these antimicrobial agents observed attached to the bacterial surface. In contrast, the polymyxins were excreted into the culture media [6]. Utilizing the antimicrobial agents produced by *P. polymyxa* in the pharmaceutical industry for clinical applications requires overcoming challenges related to achieving pure and large-scale production of antibiotics. In a study by Chen et al. (2024), metabolic engineering was employed on *P. polymyxa* ATCC842 to engineer the L-2,4-diaminobutyric acid pathway, resulting in the creation of *P. polymyxa* P19. This engineered strain exhibited a remarkable 269% increase in the production of polymyxin E (colistin). By simultaneously supplementing L-isoleucine and L-leucine, the purity of the antibiotic was enhanced, making it suitable for clinical use [54]. In a study by Li et al. (2024), a challenge encountered while using *P. polymyxa* for antibiotic production was the potential susceptibility of the bacteria to the antibiotics they produce, such as in the case of polymyxin B-induced bacterial death during production. The researchers aimed to investigate the mechanism of resistance exhibited by *P. polymyxa* SC2 when exposed to polymyxin B. Treatment with polymyxin B led to gene upregulation and physiological changes in the strain. However, the results showed that the addition of magnesium, calcium, and iron enhanced resistance to polymyxin B by reducing oxidative damage, promoting biofilm formation, and implementing other resistance mechanisms. These findings have implications for protecting the bacteria and improving the yields of polymyxins, offering insights into strategies to mitigate antibiotic-induced bacterial susceptibility during production processes [55].

In an alternative application, *P. polymyxa* may serve as a biofungicide agent, offering a sustainable substitute for chemical fungicides commonly used in agriculture. Biofungicides are typically formulated in powder form using the freeze-drying method to enhance storage stability, necessitating the use of protective agents for the bacterial cells [34]. Research by Nasran et al. (2020) identified that for *P. polymyxa* strain Kp10, an optimal mixture of lactose (10% w/v) and sucrose (27.5% w/v) served as effective protective agents, enhancing bacterial cell viability. This formulation demonstrated efficacy against fungal phytopathogens, suggesting potential for the bacterium as a biofungicide. Further experimentation would be needed to investigate its broad application as a biofungicide in agriculture [34].

Bacteriocins, natural antimicrobial peptides produced by bacteria, offer a natural alternative to chemical preservatives in food manufacturing [56]. However, achieving high yields of these compounds for commercial use presents a challenge. In a study by El-Sharoud et al. (2022), it was discovered that the production of paenibacillin, a bacteriocin generated by *P. polymyxa* strain OSY-DF, could be enhanced by subjecting the bacteria to environmental stress such as cold temperatures. The study demonstrated the efficacy of penicillin as a bio-preservative for milk [57].

Table 1 presents a summary of representative chemicals produced by *P. polymyxa*.

**Table 1.** Overview of *Paenibacillus polymyxa* strains, their chemicals, mechanisms, and effects/applications.

Strain	Application	Chemicals Produced	Mechanism of Action	Outcome	References
*P. polymyxa* MaAl70	Biofertilizer	Organic acids, Ammonia, IAA	**Methane Consumption:** Reduces greenhouse gas emissions by oxidizing methane. **Nutrient Solubilization:** Phosphorus (P): Produces organic acids to release soluble phosphate. Potassium (K): Solubilizes K from minerals like feldspar. Zinc (Zn): Mobilizes Zn for plant uptake. **Nitrogen Fixation:** Converts atmospheric nitrogen into ammonia, enriching soil fertility.	Enhanced crop yield and soil health, and reduced chemical fertilizer use and methane emissions.	[35]
*P. polymyxa* CAPE238	Biofertilizer	Phytohormones (e.g., IAA)	**Enhanced Seed Germination:** Produces growth-promoting substances **Plant Growth Promotion:** Nutrient Solubilization Nitrogen Fixation **Stress Mitigation:** May produce stress-alleviating compounds to improve plant resilience.	Improved crop yield, faster germination, and healthier plant growth.	[36]
*P. polymyxa* PKB1	Biocontrol agent	Antifungal peptides (e.g., fusaricidins)	**Antifungal Peptides:** disrupt the cell membranes of phytopathogenic fungi, leading to cell lysis and death. **Broad-Spectrum Activity:** Effective against a wide range of phytopathogens, including *Fusarium*, *Aspergillus*, and *Botrytis*.	Suppression of fungal pathogens, improved crop health, and enhanced yield.	[37]
*P. polymyxa* 3-3	Biotechnology: Production of KGM.	Glucomannanase	**Glucomannanase** hydrolyzes KGM into oligosaccharides.	Sustainable production of valuable oligosaccharides with applications in food, health, and biotechnology sectors.	[39]
*P. polymyxa* strain 26	Waste Management	Laccase, Lignin peroxidase, Manganese peroxidase	**Ligninolytic Enzymes Production:** Laccase: Oxidizes lignin and phenolic compounds. Lignin Peroxidase: Breaks down lignin polymers. Manganese Peroxidase: Degrades lignin and detoxifies pollutants.	Sustainable waste management, reduced environmental impact, and production of valuable byproducts like biofuels and compost.	[42]
*P. polymyxa* SCE2	Cleaning and disposal of environmental waste.	Organic acids, Enzymes (e.g., proteases, lipases, cellulases)	**Microbially Induced Corrosion (MIC):** Produces metabolites that degrade complex waste materials, including metals and organic pollutants. Can accelerate corrosion of metal waste. **Biodegradation:** Secretes extracellular enzymes to degrade organic waste.	Efficient and eco-friendly waste disposal, reduced environmental pollution, and improved waste management practices.	[43]
*P. polymyxa* B-4317 and *P. polymyxa* ATCC842	Production of 2,3-BDL	Enzymes such as acetolactate synthase, acetoin reductase, and 2,3-BDL dehydrogenase.	**Bacterial Fermentation:** Converts carbon sources (2,3-BDL via metabolic pathways. **Key Pathways:** Pyruvate is converted to acetolactate, then to acetoin, and finally to 2,3-BDL.	Sustainable production of 2,3-BDL from renewable carbon sources, reducing reliance on petroleum-based chemicals.	[46], [50]
*P. polymyxa* TH2H2, DSM32871 and M1	Biocontrol Agent	Fusaricidin, Tridecaptin, Polymyxins	**Antifungal Activity:** fusaricidin, a lipopeptide that disrupts fungal cell membranes, is effective against phytopathogens like *Fusarium* and *Aspergillus*. **Antibacterial Activity:** Produces tridecaptin and polymyxins, which target Gram-negative bacteria by disrupting their cell membranes.	Improved crop protection, reduced disease incidence, and enhanced agricultural productivity.	[6], [53]
*P. polymyxa* P19	Biocontrol Agent:	Polymyxin E (colistin)	**Metabolic Engineering:** *P. polymyxa* P19 is genetically modified to enhance the production of polymyxin E. **Antibacterial Activity:** Polymyxin E disrupts the cell membranes of Gram-negative bacteria, making it effective against pathogens like *Pseudomonas aeruginosa* and *E. coli*.	Enhanced polymyxin E production for effective biocontrol and medical applications, contributing to sustainable agriculture and combating antibiotic resistance.	[54]
*P. polymyxa* OSY-DF	Bio-preservative	Paenibacillin	**Paenibacillin:** Paenibacillin, a potent antimicrobial peptide effective against Gram-positive bacteria, including foodborne pathogens like *Listeria* and *Staphylococcus*. **Cold Temperature Enhancement:** Exposure to cold temperatures (e.g., 10–15°C) enhances penicillin production, making it suitable for applications in refrigerated food preservation.	Enhanced food safety, extended shelf life, and reduced food waste through natural bio-preservation.	[57]

### 2.2. Application of *P. polymyxa* in agriculture

In addition to the discussed agricultural applications of *P. polymyxa*, further aspects of its utilization in agriculture will be explored. The rhizosphere microbiome, where *P. polymyxa* resides, is vital in enhancing plant growth, protecting plants from environmental stress, and improving nutrient uptake. *P. polymyxa* has been found to elevate plant nitrogen and iron levels, contributing to its PGP characteristics [58]. Furthermore, gene sequencing studies of *P. polymyxa* DMS 365 have revealed the presence of genes encoding enzymes related to carbohydrate metabolism, nitrogen fixation, and siderophore biosynthesis. This genetic repertoire underscores the significance of *P. polymyxa* in promoting plant growth and its potential applications in the industrial sector [59]. The multifaceted capabilities of *P. polymyxa* highlight its significance in agriculture and industry, emphasizing its role in enhancing plant health, nutrient uptake, and overall agricultural productivity.

**Figure 2.**
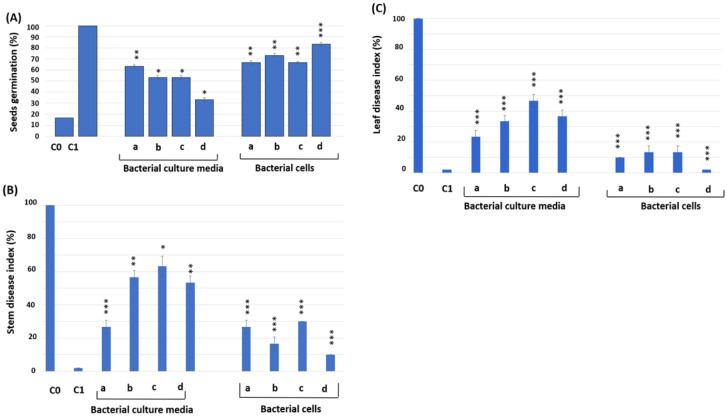
Seeds germination (A), stem disease index (B), and leaf disease index (C) (±SE) of wheat (cultivar Om Rabiia) grown in pots with the phytopathogenic fungus *F. graminearum* and the antagonist bacteria. Fg: *F. graminearum*. C0: Wheat growing with Fg but without BLB369, BLB277, and BLB267; C1: wheat growing without Fg, BLB369, BLB277, and BLB267. (a) BLB267 + Fg; (b) BLB369 + BLB267 + Fg; (c) BLB277 + BLB267 + Fg; (d) BLB369 + BLB277 + BLB267 + Fg. The level of significance was determined in comparison with the control group C0. ***Significance level 99.9%; **significance level 99%; and *significance level 95% [60].

In a study assessing the impact of *P. polymyxa* strain BLB267 on wheat seed germination and resistance to *Fusarium* wilt infestation under greenhouse conditions, it was found that this strain is compatible and significantly enhances seed germination while reducing disease incidence. When used alone or in combination with *Bacillus amyloliquefaciens* BLB369 and *B. subtilis* BLB277, the strain showed positive effects. The bacterial culture media led to a germination rate of 33–63%, whereas direct application of bacterial cells or their combinations resulted in a higher germination rate of 66–83%. In contrast, control seeds inoculated with *Fusarium graminearum*, without bacterial treatment, exhibited less than 18% germination. Seeds that were not exposed to the fungus and did not receive bacterial treatment showed 100% germination. Moreover, the treated wheat plantlets demonstrated substantial reductions in *Fusarium* wilt, with whole bacterial cells offering better protection (70–90% for stems and 87–98% for leaves) compared to the culture media alone. The application of *P. polymyxa* BLB267 showcased high levels of protection, indicating its potential as an effective and sustainable biocontrol agent [60]. This research highlights the beneficial effects of *P. polymyxa* in promoting seed germination and providing protection against fungal pathogens in wheat plants (Figure 2).

Moreover, research conducted by Weselowski et al. (2016) showed that *P. polymyxa* CR1 has several advantageous characteristics significant for sustainable agriculture. This strain showed antagonistic properties against prevalent plant pathogens like *Phytophthora sojae*, *Rhizoctonia solani*, and *Cylindrocarpon destructans*. It also promoted the growth of several plants, such as maize, potatoes, cucumbers, Arabidopsis, and tomatoes, by leveraging atmospheric nitrogen and insoluble phosphorus, generating the phytohormone IAA, and breaking down lignocellulosic components [15].

Rhizosheath, the important soil layer surrounding plant roots, is crucial in supporting plants during drought conditions. The formation of the rhizosheath is influenced by interactions among root exudates, microbes, and soil conditions, with development being more pronounced in acidic soils than alkaline soils [61]. Research has demonstrated that *P. polymyxa*, which produces the plant growth hormone IAA, enhances rhizosheath formation in *Hordeum vulgare* L., leading to improved cereal yields. Inoculating alkaline soil with *P. polymyxa* isolated from acidic soil has been shown to increase rhizosheath formation in the alkaline soil, thereby emphasizing the role of *P. polymyxa* in promoting rhizosheath development for enhancing crop yields and sustainability [62]. Similarly, another strain of *P. polymyxa*, *P. polymyxa* ZYPP18, has exhibited plant growth promotion and biocontrol activities against *Rhizoctonia cerealis*, a wheat sheath blight pathogen. This strain exerts its effects through the production of IAA for plant growth promotion and antimicrobial agents for biocontrol purposes. The study underscores the multifaceted agricultural benefits of *P. polymyxa* in enhancing plant growth and defense mechanisms against plant pathogens [63].

*P. polymyxa* has displayed remarkable potential as a biocontrol agent, offering a sustainable alternative to chemical interventions in agriculture for plant disease management [34]. One significant challenge in kiwifruit cultivation is kiwifruit bacterial canker caused by *Pseudomonas syringae* pv. *actinidiae*. A study revealed that *P. polymyxa* strain YLC1 exhibits antagonistic activity against this pathogen in field trials. Polymyxin B1 was identified as the bioactive compound responsible for this activity, underscoring the outstanding biocontrol effects of *P. polymyxa* YLC1 [64]. In another study, *P. polymyxa* strain J2-4 demonstrated biocontrol activity against *Meloidogyne incognita*, a devastating cucumber nematode pathogen, by inhibiting pathogen invasion and development. Genes encoding antimicrobial compounds such as polymyxin, fusaricidin B, paenilan, and tridecaptin were identified in this strain, while no genes encoding virulence factors were found. This highlights the potential of *P. polymyxa* J2-4 as a non-pathogenic biocontrol agent in agriculture, offering effective management against plant nematode pathogens [65].

Additionally, in a study by Abdelkhalek et al. (2022) *P. polymyxa* strain SZYM exhibited antiviral activity against zucchini yellow mosaic virus in greenhouse conditions. *P. polymyxa* SZYM was found to enhance the growth of treated plants and provide protection against the virus by reducing virus accumulation and upregulating defense-related genes in the plants. Furthermore, the bacterium led to an increase in the levels of reactive oxygen species scavenging enzymes and a decrease in non-enzymatic oxidative stress markers, indicating its role in enhancing plant defense mechanisms and mitigating oxidative stress [66]. This research highlights the potential of *P. polymyxa* as a beneficial biocontrol agent with antiviral properties that can contribute to plant health and protection from viral infections.

*P. polymyxa* has demonstrated its capability to mitigate environmental stress and enhance plant growth. In a study involving maize (*Zea mays* L.) seeds coated with *P. polymyxa* and exposed to elevated copper concentrations to induce copper stress, the bacterium effectively alleviated the copper stress through various mechanisms. *P. polymyxa* improved the osmolyte content in maize, assisting the plant in maintaining optimal water balance, and reduced the accumulation of proline, leading to enhanced plant growth. Additionally, the bacterium played a role in reducing the accumulation of copper in the roots and shoots of the plants, while also boosting the antioxidant defense system. This enhancement in the reactive oxygen species scavenging capacity protected the plants from oxidative damage caused by copper-induced stress [67]. These findings underscore the potential of *P. polymyxa* in promoting plant resilience to environmental stress and improving growth under challenging conditions.

Soil salinity presents a significant environmental stress that can impede plant growth and yield. *P. polymyxa* strain SC2 has shown a salt-tolerance trait attributed to its capacity to synthesize exopolysaccharides. These exopolysaccharides assist in sequestering toxic heavy metals, metabolizing purine and pyrimidine, and facilitating the transport of osmoprotectants like potassium [68]. In a study by Wang et al. (2024), three mutants (*SC2-11*, *SC2-13*, and *SC2-14*) exhibiting improved salt tolerance effects were isolated. The researchers' aimed to unravel the genetic traits underlying this enhanced salt tolerance, providing valuable insights that could potentially be leveraged for utilizing *P. polymyxa* against salinity stress [68]. This research highlights the potential of *P. polymyxa* in mitigating the detrimental effects of soil salinity on plants by enhancing salt tolerance through genetic modifications.

In a study by Jozay et al. (2024), irrigating plants with recycled wastewater led to increased levels of proline and phenols, which act as non-enzymatic antioxidants to counteract the oxidative stress induced by the water. To address this, a rhizobacteria mixture was employed as a biofertilizer on plants irrigated with recycled wastewater. The study demonstrated that the mixture, which included *P. polymyxa* along with other rhizobacteria, effectively reduced oxidative damage by enhancing the production of antioxidants. This approach showcased the potential of using beneficial rhizobacteria, such as *P. polymyxa*, to mitigate oxidative stress in plants irrigated with recycled wastewater, thereby promoting plant health and productivity [69].

Table 2 offers a comprehensive overview of the diverse agricultural applications of *P. polymyxa*.

**Table 2.** Agricultural applications of *Paenibacillus polymyxa* strains: Chemicals, mechanisms, and benefits.

Strain	Application	Chemicals Produced	Mechanism of Action	Outcome	Reference
*P. polymyxa* DMS 365	PGP	Simple sugars, Ammonia, and Iron-chelating compounds	**Carbohydrate Metabolism** **Nitrogen Fixation** **Siderophore Biosynthesis:** Produces iron-chelating compounds.	Improved plant growth and soil fertility	[59]
*P. polymyxa* ZYPP18	PGP Biocontrol	IAA, Antimicrobial agents	**IAA Production:** IAA promotes root growth and plant development. **Antimicrobial Agents:** compounds that inhibit pathogens.	Enhanced wheat growth and yield, reduced need for chemical pesticides	[63]
*P. polymyxa* YLC1	Biocontrol	Polymyxin B1	**Polymyxin B1 Production:** Produces polymyxin B1, which disrupts the cell membranes of Gram-negative bacteria like *Pseudomonas syringae*.	Protection of crops from bacterial pathogens	[64]
*P. polymyxa* J2-4	Biocontrol agent	Polymyxin, Fusaricidin B, Paenilan, Tridecaptin	**Strain-specific antimicrobials:** J2-4 produces polymyxin (targets Gram-negative bacteria), fusaricidin B (directly disrupts nematode membranes and eggshells), paenilan, and tridecaptin, which synergistically weaken nematode survival and reproduction. **Microbiome modulation:** Suppresses soil bacteria/fungi that support *Meloidogyne incognita* virulence (e.g., nutrient scavengers or root-attacking symbionts).	Suppression of nematode survival and reproduction, improved crop health	[65]
*P. polymyxa* SZYM	Antiviral activity	Antiviral compounds, ROS scavenging enzymes	**Direct antiviral activity:** Produces compounds that inhibit viral replication or spread. **Induced systemic resistance (ISR):** Activates the plant's immune system, enhancing defense against viruses. **Antioxidant activity:** Boosts ROS scavenging enzymes (e.g., SOD, CAT, POD) and reduces oxidative stress markers, protecting plants from virus-induced damage.	Enhanced plant defense against zucchini yellow mosaic virus, reduced virus-induced damage	[66]
*P. polymyxa* SC2	Salt-tolerance trait	Exopolysaccharides, Osmoprotectants	Synthesize exopolysaccharides to sequester heavy metals Metabolize purine and pyrimidine Transportation of osmoprotectants.	Improved plant tolerance to salt stress	[68]

### 2.3. Medical application of *P. polymyxa*

Antibiotic resistance poses a significant challenge in the treatment of infections, with limited alternatives available. Antimicrobial peptides derived from *P. polymyxa* and other bacteria offer a potential solution [70].

*P. polymyxa* produces antimicrobial peptides such as polymyxin B and polymyxin E (colistin), known for their potent bactericidal activity against Gram-negative bacteria by disrupting the bacterial cell membrane. While polymyxin B and polymyxin E are utilized clinically as last-resort antibiotics, reports of resistance to these drugs have emerged as a concern [71]. In a study, *P. polymyxa* strain HS5 was found to produce epsilon-poly-l-lysine, an antimicrobial peptide showing strong activity against various pathogens, including strains resistant to polymyxins. This discovery highlights epsilon-poly-l-lysine as a promising alternative antimicrobial agent with potential efficacy against resistant strains [72]. This research underscores the importance of exploring novel antimicrobial agents from *P. polymyxa* to combat antibiotic resistance and address the evolving challenges in infection treatment.

*P. polymyxa* strain Kp10 has been shown to exhibit antimicrobial activity against *Listeria monocytogenes*, a foodborne pathogen. The anti-listerial activity of *P. polymyxa* Kp10 was attributed to histone-like DNA-binding proteins, translation initiation factor IF-1, and 50S ribosomal protein L29. Additionally, the study suggested that other proteins produced by Kp10 may also contribute to its antimicrobial effects [73]. This research highlights the potential of *P. polymyxa* as a biocontrol agent against foodborne pathogens, showcasing its antimicrobial properties and the diverse mechanisms underlying its activity.

In chronic wound infections, biofilms can pose a significant challenge as they make it difficult for antibiotics to effectively penetrate and treat the infection. One strategy for managing these infections involves the use of anti-biofilm peptides such as bacteriocins and protease enzymes [74]. In a docking analysis conducted by Ghoreishi et al. (2022), the metalloprotease produced by *P. polymyxa* was observed to interact with proteins involved in biofilm development by common wound infection pathogens like *Staphylococcus aureus* and *Pseudomonas aeruginosa*. This interaction suggests that the metalloprotease has the potential to disrupt the biofilm structure and aid in its destruction, offering a promising approach to combating biofilms in chronic wound infections [75].

Zhang et al. (2020) have innovatively developed microneedles as a drug delivery system, overcoming challenges associated with drug administration. These microneedles are designed to conform well to the skin, exhibit strong adhesion, and are loaded with polymyxin B, an antimicrobial agent, to reduce the risk of infection while effectively delivering the drug [76]. This novel approach showcases the potential of microneedles as a precise and efficient drug delivery method, offering targeted antimicrobial therapy with enhanced patient comfort and reduced infection risk.

In a recent study by Xie et al. (2024), the anti-inflammatory properties of the exopolysaccharide EPS1, secreted by *P. polymyxa*, were investigated in the context of fungal infection caused by *Malassezia restricta*. The effects of EPS1 were studied both in vitro and in a murine model. The results showed that EPS1 improved the skin condition, reduced proinflammatory responses, enhanced the function of regulatory T cells (Tregs), and modulated the normal skin flora. This research suggests that EPS1 has the potential to serve as a therapeutic agent for the treatment of skin damage induced by *Malassezia*, highlighting the promising anti-inflammatory and protective effects of this exopolysaccharide derived from *P. polymyxa* [77].

In a study by Yasuzawa et al. (2022), Enzamin, a product derived from *P. polymyxa* AK, demonstrated anti-inflammatory and intestinal microbiota modulation activities. In an experiment involving mice fed a high-fat diet, Enzamin was found to alleviate adipose inflammation by reducing adipocytokine expression and insulin resistance. Additionally, Enzamin was observed to enhance the population of intestinal microbiota, which likely contributed to its role as an inflammatory regulator in obese mice. These findings suggest that Enzamin has potential as a therapeutic agent for managing inflammation associated with obesity, with its beneficial effects attributed to its impact on adipose tissue and modulation of intestinal microbiota [78].

*P. polymyxa* S4 produces pectinase, an enzyme that exhibits significant antioxidant activity. The byproducts of pectinase activity, including pectic oligosaccharides and pectic polysaccharides, have demonstrated potent antitumor properties without inducing toxicity to living cells. These characteristics highlight the potential of these compounds as promising bioactive agents. Additionally, the enzyme's ability to degrade fruit waste further underscores its utility in sustainable applications [38].

Table 3 offers a comprehensive overview of the diverse medical applications of *P. polymyxa*.

**Table 3.** Medical and health applications of *Paenibacillus polymyxa* strains: Chemicals, mechanisms, and benefits.

Strain	Application	Chemicals Produced	Mechanism of Action	Outcome	Reference
*P. polymyxa* HS5	Antimicrobial agent	Epsilon-poly-I-lysine	Production of epsilon-poly-I-lysine an antimicrobial agent against a range of pathogens, including some polymyxin-resistant strains	Effective control of various pathogens	[72]
*P. polymyxa* Kp10	Antimicrobial agent	Antimicrobial proteins	Production of proteins with antimicrobial activity against *Listeria monocytogenes*	Enhanced food safety	[73]
*P. polymyxa* AK	Anti-inflammatory and intestinal microbiota modulator	Enzamin	Production of enzamin, which weakened adipocytokine expression and insulin resistance Enhanced the intestinal microbiota population in obese mice	Improved metabolic health and gut microbiota balance	[78]
*P. polymyxa* S4	Antioxidant and antitumor activity	Pectinase, Pectic oligosaccharides, Pectic polysaccharides	Production of pectinase with high antioxidant activity pectic oligosaccharides and pectic polysaccharides byproducts have antitumor properties	Enhanced antioxidant defense, potential antitumor effects	[38]

## 3. Genetic manipulation of *P. polymyxa*

Recent advancements in the genetic manipulation of *P. polymyxa* have introduced a simplified method for gene knockout and direct screening of recombinant clones, significantly facilitating targeted mutagenesis [12]. This system leverages homologous recombination enhanced by suicide plasmid denaturation, enabling the disruption of specific genes such as the α- and β-amylase genes [12]. The successful inactivation of these genes serves as a useful genetic marker for screening and opens avenues for functional studies of other genes contributing to the beneficial traits of this bacterium [12]. Additionally, the establishment of inducible expression systems using compatible theta- and rolling-circle replicating vectors has expanded the genetic toolbox available for *P. polymyxa*, enabling controlled gene expression essential for functional genomic studies [79].

Research has also highlighted the use of CRISPR-Cas9 technology for precise genome editing in *P. polymyxa*, enabling efficient deletions and insertions [9],[80]. Despite these developments, challenges remain, particularly concerning transformation efficiency and subsequent homologous recombination events, which may be less effective than desired. However, ongoing research aims to optimize these systems to enhance their application potential in biotechnological fields [81]. Overall, the genetic manipulation and gene expression profiling in *P. polymyxa* present a promising frontier that can significantly impact fundamental microbiological research and practical agricultural applications.

### 3.1. Mutagenesis techniques

Ethyl methanesulfonate (EMS) and N-methyl-N′-nitro-N-nitrosoguanidine (MNNG) are commonly used chemical mutagens to induce mutations in bacterial genomes [82]. These agents have been utilized in studies, such as the mutagenesis of *Bacillus* spp. using EMS for improved enzyme activity [83] and the use of EMS and MNNG in yeast and other model organisms [84]. These mutations can lead to phenotypic variations that may enhance plant growth-promoting characteristics or biocontrol abilities [85].

In another recent investigation, physical agents, such as ultraviolet (UV) radiation or ionizing radiation, were used to induce mutations. UV radiation causes DNA damage, leading to mutations during repair processes. This method has been utilized to isolate *P. polymyxa* strains with improved traits, such as increased antibiotic production [15].

Furthermore, *P. polymyxa* has a natural competence for transformation, enabling it to take up exogenous DNA from its environment. This process can be harnessed to introduce plasmids or linear DNA fragments into the bacterial genome. Natural transformation is often facilitated by specific growth conditions, such as nutrient-rich media or stationary phase growth [86].

In addition, *P. polymyxa* is recognized for its ability to enhance nutrient availability, particularly nitrogen and phosphorus, in the rhizosphere [15]. Genetic manipulation has enabled the optimization of metabolic pathways involved in nutrient solubilization and plant hormone production, resulting in the development of more effective biofertilizer formulations [5]. These biofertilizers can improve soil fertility and promote sustainable agricultural practices by reducing the reliance on chemical fertilizers. Genetic alterations have been utilized to improve its ability to fix nitrogen and produce plant growth-promoting compounds, thereby supporting sustainable agricultural practices [87].

Genetic manipulation techniques have been employed to enhance the biocontrol capabilities of *P. polymyxa* against plant pathogens. By manipulating genes related to the production of antagonistic compounds, such as fusaricidins, researchers aim to enhance the bacterium's efficacy in suppressing plant diseases [9]. For instance, utilizing PCR-targeted mutagenesis has been effective in disrupting the biosynthesis of these antifungal antibiotics [9]. By introducing or enhancing genes responsible for the production of antimicrobial compounds, researchers have developed strains that exhibit increased resistance to soil-borne diseases such as *Fusarium* wilt and root rot [88],[89]. These modified strains suppress pathogens, promote plant resilience, enhance plant growth, and promote root colonization, which is crucial for biocontrol against plant pathogens like *Phytophthora palmivora* and improving crop yields [9].

### 3.2. Plasmid-based transformation and genetic engineering methods in *P. polymyxa*

Plasmid vectors are widely utilized to introduce foreign genes into *P. polymyxa*, enabling genetic manipulation and the expression of desirable traits. These vectors typically carry selectable markers, such as antibiotic resistance genes, which facilitate the identification of successfully transformed cells. Common plasmid-based transformation methods include electroporation and heat shock, both of which enhance the uptake of plasmid DNA into bacterial cells [86]. Among these, electroporation has been particularly optimized for *P. polymyxa*. This technique involves applying an electric field to create transient pores in the cell membrane, enabling exogenous DNA to enter the cells efficiently [90]. In addition to electroporation, conjugation has emerged as a valuable method for genetic engineering in *P. polymyxa*. Conjugation involves the direct transfer of plasmid DNA from one bacterium to another through cell-to-cell contact, enabling the introduction of plasmids carrying beneficial traits from other bacterial species. This approach not only broadens the genetic diversity of *P. polymyxa* but also enhances its potential applications in agriculture, industry, and biotechnology [91].

### 3.3. Gene knockout and functional analysis in *P. polymyxa*

Gene knockout techniques have become indispensable tools for elucidating the functional roles of specific genes in *P. polymyxa*. By disrupting target genes, researchers can investigate their contributions to the bacterium's metabolic pathways, stress responses, and interactions with plants and other microorganisms [9]. Advancements in genetic engineering, particularly the application of CRISPR-Cas9 and homologous recombination, have significantly enhanced the precision and efficiency of gene knockout in *P. polymyxa* [10],[92]. The CRISPR/Cas9 system has revolutionized genetic manipulation by allowing precise editing of specific genomic loci. This technique was employed to knock out undesirable genes or introduce beneficial traits into *P. polymyxa*. For example, researchers have successfully used CRISPR/Cas9 to enhance the production of secondary metabolites and improve biocontrol efficacy against plant pathogens [90].

*P. polymyxa* is known for producing a variety of secondary metabolites with antimicrobial and antifungal properties. Genetic manipulation techniques, such as CRISPR/Cas9 and plasmid-based expression systems, have been employed to enhance the production of these valuable metabolites [12]. By optimizing the biosynthetic pathways through targeted gene editing, researchers have been able to increase yields of compounds such as polymyxin and other bioactive substances, which have potential applications in pharmaceuticals and agriculture. The bacterium's ability to produce polymyxins, valuable antibiotics, has been further optimized through genetic manipulation. By introducing foreign genes and disrupting existing ones, such as the *abr*B gene in a heterologous system, researchers have been able to increase polymyxin production significantly [93].

#### 3.3.1. CRISPR-Cas9 and homologous recombination

The implementation of a CRISPR-based system for efficient genome editing in *P. polymyxa* was first reported by the researchers in [79]. This single-vector-based system enabled both single-gene deletions and the removal of an 18 kb genomic region using two targeting sgRNAs, demonstrating its versatility for precise genetic modifications [94]. Additionally, a gene regulation system for single and multiple targets was developed using the nuclease-deactivated variant of an optimized Cas12a (dCas12a) from *Acidaminococcus* sp. [95]. Further advancements include the development of a multiplex base editing tool by Kim et al. (2021), which combines dCas9 with a cytidine deaminase to mediate C-to-T substitutions, expanding the genome editing toolkit for *P. polymyxa* [96].

Despite these innovations, multiplex gene deletions or integrations have not been achieved in *P. polymyxa*. The ability to perform such modifications is highly desirable for metabolic engineering and the development of chassis organisms, as it allows for the simultaneous alteration of multiple genes or pathways. In this study, researchers focused on exploiting multiplex genome editing to enable simultaneous gene deletions and integrations in *P. polymyxa* [10]. Genes and clusters essential for EPS production were selected as initial targets to facilitate straightforward screening [12]. Furthermore, researchers demonstrated the functionality of a single sgRNA-guided CRISPR-Cas9 approach for deleting large genomic clusters, a critical step toward creating mutant strains with minimized genomes [96]. These efforts aim to develop a robust and versatile chassis organism for biotechnological applications [94].

CRISPR-Cas9 has revolutionized gene editing in *P. polymyxa* by enabling targeted gene disruptions with high specificity. This system enables the creation of isogenic mutants, which are critical for functional analysis. For example, CRISPR-Cas9 has been used to knockout genes involved in nitrogen fixation, biofilm formation, and secondary metabolite production, providing insights into their roles in plant growth promotion and stress tolerance [97]. Homologous recombination, another widely used technique, involves the replacement of target genes with selectable markers, such as antibiotic resistance genes, to facilitate the identification of successful knockouts. This method has been employed to study genes encoding hydrolytic enzymes, such as cellulases and chitinases, which are essential for *P*. *polymyxa*'s biocontrol capabilities [98].

In *P. polymyxa*, researchers have successfully implemented nuclease-deactivated Cas12a (dCas12a) to develop a CRISPR interference/activation (CRISPRi/a) tool for targeted transcriptional perturbation of both single and multiple genes [99]. This system enables precise modulation of gene expression, providing a powerful approach for functional genomics studies [12]. However, attempts to utilize the endonuclease activity of Cas12a for genome editing in *P. polymyxa* have not yielded positive results (unpublished data) [96].

Additionally, a base editing tool, which combines dCas9 with a cytidine deaminase for multiplex genome editing, has been developed by Kim et al. (2021), offering a promising avenue for precise nucleotide-level modifications [96]. Despite these advancements, tools for large genomic deletions and multiplex genomic modifications remain unexplored in *P. polymyxa*, highlighting a critical gap in the current genetic engineering toolkit for this species [99]. In this study, they demonstrated the versatility of a Cas9-based system in *P. polymyxa* for single-gene deletions, large cluster deletions, and multiplex genome editing [12]. Targeting *sgRNA*s closer to the middle or 3′ end of biosynthetic gene clusters (*BGC*s) enhanced deletion efficiency, enabling the removal of 45 kb from the genome [10]. Achieving multiplex gene integrations in *P. polymyxa* represents a significant milestone, as this capability is rarely accomplished in bacterial systems [96]. These findings highlight the system's potential for genome reduction and strain development, though further optimization is needed for multiplex *BGC* replacements [99]. This Cas9-based tool is expected to advance genetic engineering in *P. polymyxa* and related species.

#### 3.3.2. Functional analysis of knockout mutants

Functional analysis of knockout mutants has revealed the importance of specific genes in *P*. *polymyxa*'s beneficial traits. For instance, the disruption of genes involved in polyketide synthase (PKS) and non-ribosomal peptide synthetase (NRPS) pathways has demonstrated their critical role in the production of antimicrobial compounds, such as polymyxins and fusaricidins [100]. Similarly, knockout studies targeting genes associated with EPS biosynthesis have highlighted their contribution to biofilm formation and root colonization, which are essential for the bacterium's plant growth-promoting effects [101]. While the functions of many genes in the *P. polymyxa* genome can be inferred from sequence homology, the roles of the majority remain uncharacterized. To address this gap, Seong-Bin Kim et al. (2013) developed a genetic tool for *P. polymyxa* that enables efficient and cost-effective creation of gene knockouts. This system utilizes marker exchange mutagenesis, facilitated by homologous recombination in competent *P. polymyxa* cells, with enhanced recombination efficiency achieved through the denaturation of suicide plasmid DNA. To validate this approach, researchers targeted the α- and β-amylase genes for disruption, integrating chloramphenicol or erythromycin resistance markers into the suicide plasmid pGEM7Z-f+ (Promega), flanked by sequences homologous to the target genes. This method represents the first reported use of gene replacement via homologous recombination in *P. polymyxa*. The α- and β-amylase genes not only served as effective targets for gene disruption but also provided a valuable genetic marker for the rapid and direct screening of recombinant clones. Although we focused on the insertional mutagenesis of the α- and β-amylase genes, the technique is broadly applicable for the mutagenesis of various genes in *P. polymyxa*. The ability to generate isogenic mutants deficient in specific functions will be instrumental in elucidating the genetic basis of *P. polymyxa*'s beneficial traits, such as its plant growth-promoting and biocontrol properties. This genetic tool thus provides a versatile platform for functional genomics and biotechnological applications in *P. polymyxa* [9].

In another study, Rütering et al. (2017) used a vector system based on CRISPR-Cas9 in *P. polymyxa*, allowing for quick and accurate genome editing. Researchers could assign potential roles to a number of genes involved in the biosynthesis of EPS by using this system to study the biosynthesis machinery for EPS production. The EPS variations produced by the CRISPR-Cas9 induced knockout studies had different monomer compositions and rheological characteristics from the wild-type polymer, which greatly advanced our knowledge and ability to use *P. polymyxa* in a variety of applications [79].

### 3.4. Challenges and limitations in the genetic manipulation of *P. polymyxa*

Despite the significant potential of genetic manipulation in *P. polymyxa* for agricultural and environmental biotechnology, several challenges and limitations must be addressed to fully harness its capabilities. One major obstacle is ensuring genetic stability and consistent expression of introduced traits. Genetic modifications can inadvertently lead to unintended mutations or genomic rearrangements, which may compromise the stability of desired traits across generations [31]. Additionally, the expression of foreign genes is often influenced by the host's regulatory mechanisms and environmental conditions, resulting in variable phenotypic outcomes that complicate the reliable application of engineered strains in agriculture [102]. Regulatory hurdles further complicate the deployment of genetically modified *P. polymyxa* strains. The use of genetically modified organisms (GMOs) in agriculture is subject to stringent and often inconsistent regulatory frameworks across different countries. These regulations typically require extensive safety and efficacy testing, along with detailed documentation, before commercialization can occur [103]. Such lengthy and costly approval processes can delay the development and adoption of beneficial strains, limiting their potential to contribute to sustainable agricultural practices [104].

Beyond technical and regulatory challenges, the genetic manipulation of microorganisms raises significant ethical concerns. These include potential risks to biodiversity, ecological balance, and the unintended consequences of releasing genetically modified strains into the environment [105]. For instance, there is a risk that modified strains could disrupt local ecosystems or inadvertently contribute to the emergence of new pathogens [106]. Ethical considerations also extend to the socioeconomic impacts on traditional farming practices and smallholder farmers, who may face challenges adapting to or competing with genetically modified products [107]. Addressing these ethical concerns is essential for fostering public trust and ensuring the responsible application of genetic technologies in agriculture and beyond.

### 3.5. Future directions in the genetic manipulation of *P. polymyxa*

The future of genetic manipulation in *P. polymyxa* is poised to unlock transformative applications in agriculture, medicine, and industry, driven by advancements in synthetic biology and systems-level engineering [9]. One promising direction lies in the application of cutting-edge gene-editing technologies, such as CRISPR/Cas9 and base editing, which enable precise modifications and the design of novel metabolic pathways [92]. These tools can be leveraged to engineer *P. polymyxa* strains with enhanced capabilities, such as increased production of bioactive compounds, improved biocontrol properties, or optimized nitrogen fixation. Coupling synthetic biology with computational modeling further enhances the ability to design complex genetic circuits, ensuring more predictable and robust outcomes in engineered strains [108].

Another exciting avenue is the exploration of multi-species genetic manipulation. By studying the genetic interactions between *P. polymyxa* and other beneficial microorganisms, researchers can develop microbial consortia that synergistically enhance plant growth, nutrient availability, and disease resistance [59]. Such consortia could form the basis of next-generation biofertilizers, offering sustainable solutions for improving crop resilience and productivity in diverse agricultural systems. Beyond agriculture, *P. polymyxa* holds significant potential for applications in medicine and industry. Its ability to produce antimicrobial compounds positions it as a valuable resource for developing new antibiotics to address the growing threat of antibiotic-resistant pathogens [102]. Additionally, the metabolic versatility of *P. polymyxa* can be harnessed to produce biofuels and bioplastics, contributing to a more sustainable and circular economy [91]. As research continues to uncover the full potential of this versatile bacterium, its applications are expected to expand across multiple sectors, driving innovations that promote environmental sustainability, public health, and industrial efficiency.

## 4. Conclusions and future perspectives

*P. polymyxa* holds immense potential across applications, particularly in agriculture, biotechnology, and environmental management. As a multifunctional bacterium, it is renowned for its ability to promote plant growth, enhance soil health, and suppress plant pathogens, making it a key candidate for sustainable agricultural practices. Its production of plant growth-promoting substances, such as auxins, coupled with its nitrogen-fixing capabilities and organic matter degradation, significantly contributes to improved crop yields and soil fertility. Additionally, *P. polymyxa* is being actively explored for its role in bioremediation, particularly in the degradation of environmental pollutants. The bacterium's metabolic versatility, including its ability to produce antimicrobial compounds, offers valuable opportunities for developing natural pesticides and biocontrol agents, which can be integrated into pest management strategies. Furthermore, its potential for industrial applications, such as the synthesis of enzymes and bioactive compounds, underscores its promise in advancing green chemistry and biotechnological innovations.

Despite its potential, several challenges must be addressed to fully realize the benefits of *P. polymyxa*. A limited understanding of its mechanisms of action, inconsistent field performance due to environmental variability, and the absence of standardized formulations and strain selection hinder its widespread adoption. Additionally, concerns regarding its long-term ecological impact, safety, and regulatory approval necessitate further research. Challenges in large-scale production, market acceptance, and the risk of resistance development also pose significant barriers to its broader implementation.

To overcome these challenges, researchers should focus on elucidating the interactions between *P. polymyxa* and other microorganisms, optimizing strain performance through genetic modification, and evaluating its long-term efficacy and environmental impact. Addressing these issues will be critical for promoting the sustainable and responsible use of *P. polymyxa* across sectors. As global efforts shift toward more sustainable agricultural and industrial practices, *P. polymyxa* stands out as a valuable resource, offering innovative solutions to enhance productivity, environmental health, and economic viability.

## Use of AI tools declaration

The authors declare they have not used Artificial Intelligence (AI) tools in the creation of this article.

## References

[1] Padda KP, Puri A, Zeng Q (2017). Effect of GFP-tagging on nitrogen fixation and plant growth promotion of an endophytic diazotrophic strain of *Paenibacillus polymyxa*. Botany.

[2] Ash C, Priest FG, Collins MD (1993). Molecular identification of rRNA group 3 bacilli (Ash, Farrow, Wallbanks and Collins) using a PCR probe test. Proposal for the creation of a new genus Paenibacillus. Antonie Van Leeuwenhoek.

[3] Mahajan GB, Balachandran L (2017). Sources of antibiotics: Hot springs. Biochem Pharmacol.

[4] Tang Q, Puri A, Padda KP (2017). Biological nitrogen fixation and plant growth promotion of lodgepole pine by an endophytic diazotroph *Paenibacillus polymyxa* and its GFP-tagged derivative. Botany.

[5] Padda KP, Puri A, Chanway CP, Meena VS, Mishra PK, Bisht JK, Pattanayak A (2017). *Paenibacillus polymyxa*: A prominent biofertilizer and biocontrol agent for sustainable agriculture. Agriculturally Important Microbes for Sustainable Agriculture.

[6] Mülner P, Schwarz E, Dietel K (2021). Fusaricidins, Polymyxins and volatiles produced by *Paenibacillus polymyxa* strains DSM 32871 and M1. Pathogens.

[7] Silva Dias BH, Jung SH, Castro Oliveira JVD (2021). C4 bacterial volatiles improve plant health. Pathogens.

[8] Pandey AK, Barbetti MJ, Lamichhane JR (2023). Paenibacillus polymyxa. Trend Microbiol.

[9] Kim SB, Timmusk S (2013). A simplified method for gene knockout and direct screening of recombinant clones for application in *Paenibacillus polymyxa*. Plos ONE.

[10] Meliawati M, Teckentrup C, Schmid J (2022). CRISPR-Cas9-mediated large cluster deletion and multiplex genome editing in *Paenibacillus polymyxa*. ACS Synth Biol.

[11] Zhang J, Zhao J, Fu Q (2024). Metabolic engineering of *Paenibacillus polymyxa* for effective production of 2,3-butanediol from poplar hydrolysate. Bioresour Technol.

[12] Ravagnan G, Meliawati M, Schmid J, Braman JC (2024). CRISPR-Cas9-Mediated genome editing in *Paenibacillus polymyxa*. Synthetic Biology: Methods and Protocols.

[13] Wallner A, Antonielli L, Mesguida O (2024). Genomic diversity in *Paenibacillus polymyxa*: unveiling distinct species groups and functional variability. BMC Genomics.

[14] Maggi F, Giuliodori AM, Brandi A (2024). Pangenome analysis of *Paenibacillus polymyxa* strains reveals the existence of multiple and functionally distinct *Paenibacillus* species. Appl Environ Microbiol.

[15] Weselowski B, Nathoo N, Eastman AW (2016). Isolation, identification and characterization of Paenibacillus polymyxa CR1 with potentials for biopesticide, biofertilization, biomass degradation and biofuel production. BMC Microbiol.

[16] Langendries S, Goormachtig S (2021). *Paenibacillus polymyxa*, a Jack of all trades. Environ Microbiol.

[17] Jeong H, Choi SK, Ryu CM (2019). Chronicle of a soil bacterium: *Paenibacillus polymyxa* E681 as a tiny guardian of plant and human health. Front Microbiol.

[18] Cheng W, Yang J, Nie Q (2017). Volatile organic compounds from Paenibacillus polymyxa KM2501-1 control *Meloidogyne incognita* by multiple strategies. Sci Rep.

[19] Rybakova D, Cernava T, Köberl M (2016). Endophytes-assisted biocontrol: novel insights in ecology and the mode of action of *Paenibacillus*. Plant Soil.

[20] Yegorenkova IV, Tregubova KV, Krasov AI (2021). Effect of exopolysaccharides of *Paenibacillus polymyxa* rhizobacteria on physiological and morphological variables of wheat seedlings. J Microbiol.

[21] Yuan Y, Xu QM, Yu SC (2020). Control of the polymyxin analog ratio by domain swapping in the nonribosomal peptide synthetase of *Paenibacillus polymyxa*. J Ind Microbiol Biotechnol.

[22] Zhang F, Li XL, Zhu SJ (2018). Biocontrol potential of *Paenibacillus polymyxa* against *Verticillium dahliae* infecting cotton plants. Biol Control.

[23] Galea CA, Han M, Zhu Y (2017). Characterization of the Polymyxin D Synthetase biosynthetic cluster and product profile of *Paenibacillus polymyxa* ATCC 10401. J Nat Prod.

[24] Yegorenkova IV, Fomina AA, Tregubova KV (2018). Immunomodulatory activity of exopolysaccharide from the rhizobacterium *Paenibacillus polymyxa* CCM 1465. Arch Microbiol.

[25] Zhou L, Zhang T, Tang S (2020). Pan-genome analysis of *Paenibacillus polymyxa* strains reveals the mechanism of plant growth promotion and biocontrol. Antonie van Leeuwenhoek.

[26] Daud NS, Azam ZM, Othman NZ (2023). Optimization of medium compositions and functional characteristics of exopolysaccharide from *Paenibacillus polymyxa* ATCC 824. Biocatal Agric Biotechnol.

[27] Grady EN, MacDonald J, Liu L (2016). Current knowledge and perspectives of *Paenibacillus*: a review. Microb Cell Fact.

[28] Wu Y, Cai P, Jing X (2019). Soil biofilm formation enhances microbial community diversity and metabolic activity. Environ Int.

[29] Zhou C, Guo J, Zhu L (2016). *Paenibacillus polymyxa* BFKC01 enhances plant iron absorption via improved root systems and activated iron acquisition mechanisms. Plant Physiol Biochem.

[30] Daud NS, Mohd Din ARJ, Rosli MA (2019). *Paenibacillus polymyxa* bioactive compounds for agricultural and biotechnological applications. Biocatal Agric Biotechnol.

[31] Khan MS, Gao J, Chen X (2020). Isolation and characterization of plant growth-promoting endophytic bacteria *Paenibacillus polymyxa* SK1 from Lilium lancifolium. BioMed Res Int.

[32] Mohd Din ARJ, Rosli MA, Mohamad Azam Z (2020). *Paenibacillus polymyxa* role involved in phosphate solubilization and growth promotion of *Zea mays* Under Abiotic Stress Condition. Proc Natl Acad Sci, India, Sect B Biol Sci.

[33] Li ZQ, Huang YL, Zhang J (2023). Ultrasound stimulated production of exopolysaccharide with anti-UV radiation activity by increasing cell permeability of *Paenibacillus polymyxa*. Process Biochem.

[34] Nasran HS, Mohd Yusof H, Halim M (2020). Optimization of protective agents for the freeze-drying of *Paenibacillus polymyxa* Kp10 as a potential biofungicide. Molecules.

[35] Rani V, Bhatia A, Nain L (2021). Methane utilizing plant growth-promoting microbial diversity analysis of flooded paddy ecosystem of India. World J Microbiol Biotechnol.

[36] Dal'Rio I, Lopes E dos S, Santaren KCF (2024). Co-inoculation of the endophytes *Bacillus thuringiensis* CAPE95 and *Paenibacillus polymyxa* CAPE238 promotes *Tropaeolum majus* L. growth and enhances its root bacterial diversity. Front Microbiol.

[37] Dobrzyński J, Naziębło A (2024). *Paenibacillus* as a biocontrol agent for fungal phytopathogens: is *P. polymyxa* the only one worth attention?. Microb Ecol.

[38] Ashrafian B, Hosseini-Abari A (2022). Investigation of bioactivity of unsaturated oligo‑galacturonic acids produced from apple waste by *Alcaligenes faecalis* AGS3 and *Paenibacillus polymyxa* S4 Pectinases. Sci Rep.

[39] Li K, Jiang C, Tan H (2021). Identification and characterization of a novel glucomannanase from Paenibacillus polymyxa. 3 Biotech.

[40] Liu Q, Luo L, Zheng L (2018). Lignins: Biosynthesis and Biological Functions in Plants. Int J Mol Sci.

[41] Zhang W, Wang W, Wang J (2021). Isolation and characterization of a novel laccase for lignin degradation, LacZ1. Appl Environ Microbiol.

[42] Edith Ayala-Rodríguez A, Valdés-Rodríguez S, Enrique Olalde-Mathieu V (2024). Extracellular ligninases production and lignin degradation by *Paenibacillus polymyxa*. J Gen Appl Microbiol.

[43] Parker GD, Plymale A, Hager J (2024). Studying microbially induced corrosion on glass using ToF-SIMS. Biointerphases.

[44] Tinôco D, Borschiver S, Coutinho PL (2021). Technological development of the bio-based 2,3-butanediol process. Biofuels Bioprod Bioref.

[45] Sabra W, Quitmann H, Zeng AP, Moo-Young M. (2011). 3.09-Microbial production of 2,3-Butanediol. Comprehensive Biotechnology.

[46] Joshi J, Langwald SV, Kruse O (2025). Immobilization of Paenibacillus polymyxa with biopolymers to enhance the production of 2,3-butanediol. Microb Cell Fact.

[47] Tinôco D, De Castro AM, Seldin L (2021). Production of (2R,3R)-butanediol by *Paenibacillus polymyxa* PM 3605 from crude glycerol supplemented with sugarcane molasses. Process Biochem.

[48] Zhang L, Chen S, Xie H (2012). Efficient acetoin production by optimization of medium components and oxygen supply control using a newly isolated *Paenibacillus polymyxa* CS107. J of Chemical Tech Biotech.

[49] Tinôco D, Pateraki C, Koutinas AA (2021). Bioprocess development for 2,3-Butanediol production by *Paenibacillus* strains. ChemBioEng Rev.

[50] Stoklosa RJ, Latona RJ, Johnston DB (2022). Assessing oxygen limiting fermentation conditions for 2,3-butanediol production from *Paenibacillus polymyxa*. Front Chem Eng.

[51] Tinôco D, Freire DMG (2023). Scale-up of 2,3-butanediol production by *Paenibacillus peoriae* NRRL BD-62 using constant oxygen transfer rate-based strategy. Fuel.

[52] Chen Q, Song Y, An Y (2024). Mechanisms and impact of rhizosphere microbial metabolites on crop health, traits, functional components: A comprehensive review. Molecules.

[53] Cho G, Kim DR, Jeon CW (2020). Draft genome sequence data of Paenibacillus Polymyxa strain TH2H2, isolated from a tomato flower in Korea. Data Brief.

[54] Chen N, Cai P, Zhang D (2024). Metabolic engineering of “last-line antibiotic” colistin in Paenibacillus polymyxa. Metab Eng.

[55] Li H, E WH, Zhao D (2024). Response of *Paenibacillus polymyxa* SC2 to the stress of polymyxin B and a key ABC transporter YwjA involved. Appl Microbiol Biotechnol.

[56] Singh B, Kumar N, Yadav A (2025). Harnessing the power of bacteriocins: A comprehensive review on sources, mechanisms, and applications in food preservation and safety. Curr Microbiol.

[57] El-Sharoud WM, Zalma SA, Yousef AE (2022). Inducing the production of the bacteriocin paenibacillin by Paenibacillus polymyxa through application of environmental stresses with relevance to milk bio-preservation. Int J Food Microbiol.

[58] Cunha I de CM da, Silva AVR da, Boleta EHM (2024). The interplay between the inoculation of plant growth-promoting rhizobacteria and the rhizosphere microbiome and their impact on plant phenotype. Microbiol Res.

[59] Kumar S, Ujor VC (2022). Complete genome sequence of *Paenibacillus polymyxa* DSM 365, a soil bacterium of agricultural and industrial importance. Microbiol Resour Announce.

[60] Zalila-Kolsi I, Ben Mahmoud A, Ali H (2016). Antagonist effects of *Bacillus* spp. strains against *Fusarium graminearum* for protection of durum wheat (*Triticum turgidum L*. subsp. durum). Microbio Res.

[61] Addesso R, Sofo A, Amato M (2023). Rhizosheath: roles, formation processes and investigation methods. Soil Systems.

[62] Xu F, Liao H, Yang J (2023). Auxin-producing bacteria promote barley rhizosheath formation. Nat Commun.

[63] Li X, Ma S, Meng Y (2023). Characterization of antagonistic bacteria *Paenibacillus polymyxa* ZYPP18 and the effects on plant growth. Plants.

[64] Wang H, Wang N, Tan Y (2023). YLC1: a promising antagonistic strain for biocontrol of pv., causing kiwifruit bacterial canker. Pest Manage Sci.

[65] Shi Q, Zhang J, Fu Q (2024). Biocontrol efficacy and induced resistance of *Paenibacillus polymyxa* J2-4 against *Meloidogyne incognita* infection in Cucumber. Phytopathology®.

[66] Abdelkhalek A, Al-Askar AA, Elbeaino T (2022). Protective and curative activities of *Paenibacillus polymyxa* against *Zucchini yellow* mosaic virus infestation in Squash Plants. Biology (Basel).

[67] Abdel Latef AAH, Zaid A, Abo-Baker A-BA-E (2020). Mitigation of copper stress in maize by inoculation with *Paenibacillus polymyxa* and *Bacillus circulans*. Plants (Basel).

[68] Wang C, Pei J, Li H (2024). Mechanisms on salt tolerant of *Paenibacillus polymyxa* SC2 and its growth-promoting effects on maize seedlings under saline conditions. Microbiol Res.

[69] Jozay M, Zarei H, Khorasaninejad S (2024). Exploring the impact of plant growth-promoting bacteria in alleviating stress on *Aptenia cordifolia* subjected to irrigation with recycled water in multifunctional external green walls. BMC Plant Biol.

[70] He Z, Kisla D, Zhang L (2007). Isolation and identification of a *Paenibacillus polymyxa* strain that coproduces a novel lantibiotic and polymyxin. Appl Environ Microbiol.

[71] Chiu S, Hancock AM, Schofner BW (2022). Causes of polymyxin treatment failure and new derivatives to fill the gap. J Antibiot (Tokyo).

[72] Hagh Ranjbar H, Hosseini-Abari A, Ghasemi SM (2023). Antibacterial activity of epsilon-poly-l-lysine produced by *Stenotrophomonas maltophilia* HS4 and Paenibacillus polymyxa HS5, alone and in combination with bacteriophages. Microbiology (Reading).

[73] Mokhtar NFK, Hashim AM, Hanish I (2020). The discovery of new antilisterial proteins from *Paenibacillus polymyxa* Kp10 via genome mining and mass spectrometry. Front Microbiol.

[74] Thapa RK, Kim JO, Kim J (2023). Antimicrobial strategies for topical biofilm-based wound infections: past, present, and future. J Pharm Investig.

[75] Ghoreishi FS, Roghanian R, Emtiazi G (2022). Novel chronic wound healing by anti-biofilm peptides and protease. Adv Pharm Bull.

[76] Zhang X, Chen G, Yu Y (2020). Bioinspired adhesive and antibacterial microneedles for versatile transdermal drug delivery. Research (Wash D C).

[77] Xie WY, Shen HL, Yan ZM (2024). Paenibacillus exopolysaccharide alleviates Malassezia-induced skin damage: Enhancing skin barrier function, regulating immune responses, and modulating microbiota. Int J Biol Macromol.

[78] Yasuzawa T, Nishi R, Ishitani S (2022). Effects of enzamin, a microbial product, on alterations of intestinal microbiota induced by a high-fat diet. Nutrients.

[79] Rütering M, Cress BF, Schilling M (2017). Tailor-made exopolysaccharides—CRISPR-Cas9 mediated genome editing in *Paenibacillus polymyxa*. Synth Biol.

[80] Okonkwo CC, Ujor V, Cornish K (2020). Inactivation of the levansucrase gene in *Paenibacillus polymyxa* DSM 365 diminishes exopolysaccharide biosynthesis during 2,3-butanediol fermentation. Appl Environ Microbiol.

[81] Irla M, Heggeset TMB, Nærdal I (2016). Genome-based genetic tool development for *Bacillus methanolicus*: Theta- and rolling circle-replicating plasmids for inducible gene expression and application to methanol-based cadaverine production. Front Microbiol.

[82] Chen L, Duan L, Sun M (2023). Current trends and insights on EMS mutagenesis application to studies on plant abiotic stress tolerance and development. Front Plant Sci.

[83] Soliman EAM, A.H. Aly N, Abosereh NA (2020). Mutagenic improvement of some bacillus strains for enhanced alpha amylase production. Int J Curr Microbiol App Sci.

[84] Hoyos-Manchado R, Villa-Consuegra S, Berraquero M (2020). Mutational Analysis of N-Ethyl-N-Nitrosourea (ENU) in the Fission Yeast *Schizosaccharomyces pombe*. G3 Genes Genomes Genet.

[85] Lawley PD, Thatcher CJ (1970). Methylation of deoxyribonucleic acid in cultured mammalian cells by *N*-methyl-*N*′-nitro-*N*-nitrosoguanidine. The influence of cellular thiol concentrations on the extent of methylation and the 6-oxygen atom of guanine as a site of methylation. Biochem J.

[86] Liu H, Wang J, Sun H (2020). Transcriptome profiles reveal the growth-promoting mechanisms of *Paenibacillus polymyxa* YC0136 on Tobacco (Nicotiana tabacum L.). Front Microbiol.

[87] Timmusk S, Pall T, Raz S (2023). The potential for plant growth-promoting bacteria to impact crop productivity in future agricultural systems is linked to understanding the principles of microbial ecology. Front Microbiol.

[88] Ryu CM, Kim J, Choi O (2006). Improvement of biological control capacity of *Paenibacillus polymyxa* E681 by seed pelleting on sesame. Biol Control.

[89] Wang Z, Wang S, Yang H (2024). Understanding the pathogenesis, biocontrol mechanisms, and factors influencing biocontrol effectiveness for soil-borne diseases in panax plants. Microorganisms.

[90] Yuan P, Chen Z, Xu M (2024). Microbial cell factories using *Paenibacillus* : status and perspectives. Critical Rev Biotechnol.

[91] Kumar P, Kumar V, Kumar R (2020). Fabrication and characterization of ceftizoxime-loaded pectin nanocarriers. Nanomaterials.

[92] Zhang Q, Xing C, Li S (2021). *In vitro* antagonism and biocontrol effects of *Paenibacillus polymyxa* JY1-5 against Botrytis cinerea in tomato. Biol Control.

[93] Choi SK, Park SY, Kim R (2009). Identification of a polymyxin synthetase gene cluster of *Paenibacillus polymyxa* and heterologous expression of the gene in *Bacillus subtilis*. J Bacteriol.

[94] Živič Z, Lipoglavšek L, Lah J (2025). A single vector system for tunable and homogeneous dual gene expression in *Escherichia coli*. Sci Rep.

[95] Rappleye CA (2023). Targeted gene deletions in the dimorphic fungal pathogen Histoplasma using an optimized episomal CRISPR/Cas9 system. mSphere.

[96] Kim MS, Kim HR, Jeong DE (2021). Cytosine base editor-mediated multiplex genome editing to accelerate discovery of novel antibiotics in *Bacillus subtilis* and *Paenibacillus polymyxa*. Front Microbiol.

[97] Yan D, Tajima H, Cline LC (2022). Genetic modification of flavone biosynthesis in rice enhances biofilm formation of soil diazotrophic bacteria and biological nitrogen fixation. Plant Biotechnol J.

[98] Kumar SS, Sabu A, Labrou N. (2019). Fibrinolytic enzymes for thrombolytic therapy. Therapeutic Enzymes: Function and Clinical Implications.

[99] Schilling C, Koffas MAG, Sieber V (2020). Novel prokaryotic CRISPR-Cas12a-Based tool for programmable transcriptional activation and repression. ACS Synth Biol.

[100] Li Y, Chen S (2019). Fusaricidin produced by *Paenibacillus polymyxa* WLY78 Induces systemic resistance against Fusarium Wilt of Cucumber. IJMS.

[101] He X, Li Q, Wang N (2021). Effects of an EPS biosynthesis gene cluster of *Paenibacillus polymyxa* WLY78 on biofilm formation and nitrogen fixation under aerobic conditions. Microorganisms.

[102] Liu H, Li Y, Ge K (2021). Interactional mechanisms of *Paenibacillus polymyxa* SC2 and pepper (*Capsicum annuum* L.) suggested by transcriptomics. BMC Microbiol.

[103] Ju JH, Jo MH, Heo SY (2023). Production of highly pure R,R-2,3-butanediol for biological plant growth promoting agent using carbon feeding control of Paenibacillus polymyxa MDBDO. Microb Cell Fact.

[104] Chen Z, Shen M, Mao C (2021). A type I restriction modification system influences genomic evolution driven by horizontal gene transfer in *Paenibacillus polymyxa*. Front Microbiol.

[105] Engwa G (2014). Genetic engineering on microorganism: the ecological and bioethical implications.

[106] Dawkins K, Esiobu N (2018). The invasive brazilian pepper tree (*Schinus terebinthifolius*) is colonized by a root microbiome enriched with alphaproteobacteria and unclassified spartobacteria. Front Microbiol.

[107] O'Sullivan GM, Philips JG, Mitchell HJ (2022). 20 years of legislation - how australia has responded to the challenge of regulating genetically modified organisms in the clinic. Front Med.

[108] Nielsen J, Keasling JD (2016). Engineering cellular metabolism. Cell.

